# Influence of Dietary Supplementation of Probiotic *Pediococcus acidilactici MA18/5M* During the Transition From Freshwater to Seawater on Intestinal Health and Microbiota of Atlantic Salmon (*Salmo salar* L.)

**DOI:** 10.3389/fmicb.2019.02243

**Published:** 2019-09-27

**Authors:** Alexander Jaramillo-Torres, Mark D. Rawling, Ana Rodiles, Heidi E. Mikalsen, Lill-Heidi Johansen, John Tinsley, Torunn Forberg, Elisabeth Aasum, Mathieu Castex, Daniel Lee Merrifield

**Affiliations:** ^1^Department of Basic Sciences and Aquatic Medicine, Faculty of Veterinary Medicine, Norwegian University of Life Sciences, Oslo, Norway; ^2^Aquaculture and Fish Nutrition Research Group, School of Biological and Marine Sciences, University of Plymouth, Plymouth, United Kingdom; ^3^Nofima – Norwegian Institute of Food, Fisheries and Aquaculture Research, Tromsø, Norway; ^4^BioMar AS, Trondheim, Norway; ^5^Lallemand SAS, Blagnac, France

**Keywords:** fish, microbiota, intestine, *Pediococcus acidilactici*, seawater transfer, high-throughput sequencing, antiviral response

## Abstract

The aim of this study was to assess the effect of the transfer from freshwater to seawater on the distal intestinal bacterial communities of Atlantic salmon (*Salmo salar* L.) and to evaluate the effect of dietary inclusion of *Pediococcus acidilactici* MA18/5M (at 1.19 × 10^6^ CFU/g). In this context, fish health and antiviral response were also investigated. A 12-week feeding trial was conducted in a flow-through rearing system involving 6 weeks in freshwater and 6 weeks in seawater. Fish received a control and probiotic diet. The composition of the salmon gut bacterial communities was determined by high-throughput sequencing of digesta and mucosa samples from both the freshwater and seawater stage. The main phyla detected during both freshwater and seawater stages were *Firmicutes*, *Proteobacteria*, *Fusobacteria*, and *Actinobacteria*. Significant differences were observed between the intestinal microbiota in the digesta and the mucosa. Both probiotic supplementation and the seawater transfer (SWT) had a substantial impact on the microbial communities, with most pronounced changes detected in the mucosal communities after SWT. This last finding together with a significantly higher antiviral response (*mx-1* and *tlr3* gene expression) in the distal intestine of fish fed the probiotic diet suggest a causal link between the microbiota modulation and activation of antiviral response. Feeding probiotics during the freshwater stage did not significantly increase survival after infectious pancreatic necrosis virus (IPNV) challenge after SWT, although higher survival was observed in one out of two replicate challenge tanks. In conclusion, this study demonstrated that both dietary probiotic supplementation and transfer from freshwater to seawater have an important role in modulating the bacterial communities in the distal intestine of Atlantic salmon. Furthermore, supplementation of the diet with *P. acidilactici* MA18/5M can modulate antiviral response.

## Introduction

Seawater transfer (SWT) is a crucial stage in the life of diadromous salmonids and is recognized to be a stressful stage in the life cycle of Atlantic salmon (*Salmo salar*, L). As a consequence, fish are often more susceptible to pathogens and stress during this period causing important losses for the salmon aquaculture industry (Roberts and Pearson, 2005; Aunsmo et al., 2008). According to the Norwegian Veterinary Institute, salmonid fish losses after SWT in Norway have increased in the last 5 years due to a number of issues including viral infections (Hjeltnes et al., 2018). Several viral salmon pathogens are prevalent in seawater; this is the case of infectious pancreatic necrosis virus (IPNV), infectious hematopoietic necrosis virus (IHNV), infectious salmon anemia virus (ISAV), and pancreas disease virus (PDV) (Lafferty et al., 2015; Johansson et al., 2016; Rodger, 2016). Interestingly, some studies have proposed a link within the substantial repression of the immune transcriptome, in particular of a pool of antiviral genes measured during smoltification and the early transition to seawater (Johansson et al., 2016; Karlsen et al., 2017). In addition to nutrient digestion, absorptive functions and acting as a physical and immunological barrier during the transition to seawater, it is widely recognized that the intestine plays a central role in the adaptation of fish to the new seawater habitat. Particularly, the intestine is involved in maintaining osmotic homoeostasis by desalinating absorbed seawater in order to avoid dehydration (Hoar, 1988; Grosell, 2010; Whittamore, 2012).

The intestine of fish harbors a broad consortium of different microorganisms (bacteria, viruses, yeasts, archaea, and protozoans) that have an active interaction with the intestine. Previous studies have focused on the bacterial microbiota, describing the importance of these organisms to the host including the production of enzymes, growth performance, immunity, and disease resistance (reviewed by Romero et al., 2014; Hoseinifar et al., 2017). The development of high-throughput sequencing targeting 16S rRNA gene has permitted in-depth characterization of bacterial communities in fish. In the last years several research groups have used high-throughput sequencing to investigate the effect of different factors such as diet (Schmidt et al., 2016; Gajardo et al., 2017; Booman et al., 2018), seasonal and environmental factors (Zarkasi et al., 2014, 2016), rearing conditions (Dehler et al., 2017a; Rud et al., 2017), disease (Karlsen et al., 2017), and SWT (Dehler et al., 2017b; Lokesh et al., 2018; Rudi et al., 2018; Jin et al., 2019) on intestinal bacteria microbiota in farmed Atlantic salmon. Most of the microbiota-related research in Atlantic salmon have not studied independently the intestinal digesta and mucosa. However, studies from our group comparing bacterial population between the digesta and mucosa have demonstrated that both samples harbor different bacterial communities and thus could respond differently to external factors (Gajardo et al., 2016; Gajardo et al., 2017). Lactic acid bacteria (LAB) are well-studied probionts in salmonids (Merrifield et al., 2010a), however, the potential effect they may have during the SWT phase has not yet received much attention. *Pediococcus acidilactici* MA18/5M has been used as a probiotic for marine and freshwater fish. It has been reported that the use of *P. acidilactici* MA18/5M as a dietary supplement has led to improvements in the gut health of salmonids including rainbow trout (Merrifield et al., 2010b; Hoseinifar et al., 2016) and Atlantic salmon (Abid et al., 2013; Vasanth et al., 2015). Previous research has also demonstrated that dietary supplementation of the probiotic *P. acidilactici* MA18/5M can modulate the intestinal microbiota of fish and stimulate various non-specific immunological parameters (Ferguson et al., 2010; Abid et al., 2013; Standen et al., 2013).

The aim of this study was to investigate the intestinal bacterial reassembly in the digesta and mucosa of Atlantic salmon following SWT. We also investigated the effect of probiotic supplementation with *P. acidilactici* MA18/5M on intestinal microbiota, antiviral response and the susceptibility of fish to an IPN challenge after SWT. Our main hypothesis in this study was that both water habitat and probiotic supplementation in the diet will have important effect on the intestinal microbiota of Atlantic salmon. We also wanted to investigate how the previously documented effects of this probiotic strain on gene expression profile in the distal intestine of Atlantic salmon will express at SWT.

## Materials and Methods

### Animal Husbandry

The trial was conducted at the Aquaculture Research Station, Tromsø (Norway) using a flow-through rearing system. The fish were treated according to Norwegian legislation and approved by the Food and Safety Authority under the ID project number 4986: “The use of functional feed to improve the overall performance of Atlantic Salmon smolt.” A batch of 1476 Atlantic salmon parr (IPN sensitive, AkvaGen strain, Kyrksæterøra, Norway) were reared at 6 h light/18 h darkness and 8°C (winter stimuli). The fish were sedated with Benzoak (benzocaine; 0.1 mg/ml), pan-jet marked with Alcalian blue subcutanously in the abdomen and allocated into six 500 L tanks (246 fish per tank, average weight 35 ± 3.4 g). Fish were acclimated for 1 week on a commercial diet and changing lighting conditions to a 24 h light regime and 12°C (summer stimuli). The oxygen saturation was maintained above 85% throughout the experiment. The feeding trial lasted 12 weeks involving 6 weeks in freshwater during smoltification (24 h light, 12°C) and 6 weeks in seawater (24 h light, 12°C). The smoltification process was followed by 24 h seawater (35‰) challenge tests (Blackburn, 1987) to confirm that the fish were all optimally smoltified by the time of SWT.

#### Diets and Experimental Design

Two basal iso-nitrogenous and iso-lipidic diets were formulated according to the known nutritional requirements of Atlantic salmon for freshwater and seawater stages (NRC, 2011). The diets used in each stage of the trial were similar, with modifications to the formulations to reflect the commercial practices and the different nutritional requirements of Atlantic salmon after smoltification (Table 1). Both extruded diets were produced by BioMar AD (Denmark). The trial was run in triplicate with tanks randomly allotted to each experimental group. At SWT fish in each of the six tanks were split: 60 fish per tank were transferred to the challenge unit for IPNV challenge (see section “Viral Challenge Post-seawater Transfer”), while 85 fish per tank were kept for the SW part of the feeding trial. The probiotic group was fed the same basal diet as the control but was supplemented with Bactocell^®^ (*P. acidilactici* MA18/5M) at 3 g/kg. Experimental groups were fed from Monday to Sunday to satiation using an automatic feeder for 4 h per day at a rate of 10–15% in excess of the expected feed consumption per day. The concentration of *P. acidilactici* in the probiotic diets was verified during the trial [1.19 × 10^6^ CFU/g following the methodology described by Castex (2009)].

**TABLE 1:** Composition of control and probiotic diets during freshwater and seawater stages.

	**Freshwater^1^**		**Seawater^1^**	
		
**Ingredients (%)^∗^**	**Control**	**Probiotic**	**Control**	**Probiotic**
Superprime fish meal	46.7	46.9	40.0	40.0
Hi pro soya	6.0	6.0	–	–
Soy protein concentrate	–	–	10.0	10.0
Corn gluten meal	3.0	3.0	5.0	5.0
Wheat gluten meal	10.0	10.0	3.9	3.9
Sunflower expeller	5.5	5.0	9.5	9.5
Wheat	5.5	5.5	12.0	12.0
Wheat flour	6.0	6.0	–	–
Fish oil (North Atlantic)	5.0	5.0	11.8	11.8
Rapeseed oil, Crude	11.8	11.8	5.0	5.0
Vitamineral mix	2.0	2.0	3.2	3.2
Bactocell		0.03	–	0.03
Chemical composition (%)				
Protein	49.1	48.5	45.9	46.0
Fat	22.0	21.8	22.2	21.4
Moisture	5.4	6.5	6.1	5.6
Ash	9.1	9.3	9.2	9.6

*^∗^All dietary ingredients were sourced from BioMar’s routine suppliers (not listed here for commercial reasons). ^1^Pellet size for Freshwater and Seawater was 2,8 and 3,5 mm respectively.*

#### Growth Performance

Growth performance and feed utilization were assessed by calculating the thermal growth coefficient (TGC) and specific growth rate (SGR), using the following formulas; thermal growth coefficient (TGC) = 1000 × [(final weight)1/3 − (initial weight)1/3 × (degree days)− 1, SGR, % day) = 100 × [ln (final mean weight) − ln (initial mean weight)] × days − 1.

#### Sample Collection During the Feeding Trial

Fish were sampled at the end of the freshwater stage, 1 day before SWT, and at week 12, i.e., 6 weeks after SWT. A total of 18 fish from each experimental group (6 from each tank) were sampled at each sampling point. Fish were euthanised by immersion in an overdose of Benzoak followed by the destruction of the brain. Under aseptic conditions, fish were opened by the mid-line, and the entire intestinal tract was dissected and adipose tissue removed. Only fish with digesta content throughout the intestine were sampled to ensure exposure to the diet. The distal intestine (DI) was sampled as follows: for histological analysis, approximately 5 mm of DI was excised and placed into a tube with 10% buffered formalin for 48 h and then transferred to 70% ethanol. For microbiological analysis, digesta was obtained by gentle squeezing of the DI with a sterile forceps into individual sterile 1.5 ml microcentrifuge tubes. Mucosal tissue was washed thoroughly three times with sterile phosphate-buffered saline (PBS; pH 7.3), and a 5 mm piece was excised and placed in a sterile 1.5 ml tube. Samples for microbiological analysis (digesta and mucosa) were snap-frozen in liquid nitrogen, transported on dry ice and subsequently stored at −20°C until DNA extraction. For gene expression analysis, DI mucosa samples were also immersed in RNAlater (Ambion, Carlsbad, CA, United States), transported at room temperature for 24–48 h and then stored at −80°C until RNA extraction.

### Microbiological Analyses

For analysis of the distal intestinal microbiota, the digesta from three fish per tank from each sampling were pooled (*n* = 3), whereas microbiota from the mucosa was analyzed from two individual fish per tank (*n* = 6).

#### DNA Extraction and Polymerase Chain Reaction

DNA was extracted from samples using the QIAamp^®^ Stool Mini Kit (Qiagen, Crawley, United Kingdom) following the modified protocol described by Falcinelli et al. (2015).

Polymerase chain reaction (PCR) was performed in duplicate targeting the V1–V2 hypervariable regions of the bacterial 16S rRNA gene using primers reported by Roeselers et al. (2011) as follows: forward primer 27F (5′-aga gttt gat cmt ggc tca g-3′), reverse primers 338R-I (5′-gcw gcc tcc cgt agg agt-3′) and 338R-II (5′-gcw gcc acc cgt agg tgt-3′). Primers were synthesized by Eurofins MWG (Ebersberg, Germany). All PCR reactions were performed using GeneAmp^®^ PCR System 9700 (Perkin-Elmer, San Jose, CA, United States). PCR reactions were carried out using 25 μL MyTaq^TM^ Red Mix (Bioline, United Kingdom), 0.5 μl of each primer (50 pmol/μl), 1 μl DNA template and adjusted to a final volume of 50 μl with molecular biology-grade water. Each reaction included a negative control (sterile, molecular grade water as template). A touchdown PCR was conducted at the following conditions: initial denaturation at 94°C for 7 min, then 10 cycles of 94°C for 30 s, 63°C for 30 s (decreasing 1°C every cycle) and 72°C for 30 s; this was followed by 25 cycles of 94°C for 30 s, 53°C for 30 s and 72°C for 30 s; final extension at 72°C for 10 min. The PCR products were checked for size and specificity by electrophoresis on 1.5% w/v agarose gel. The duplicate PCR reactions were combined and purified with QIAquick PCR Purification Kit (Qiagen, Valencia, CA, United States), following the manufacturer’s protocol. Purified samples were evaluated with Bioanalyzer previous to amplicon library preparation.

#### Amplicon Library and Sequencing

Prior to Ion Torrent PGM sequencing, the amplicons were assessed for fragment concentration using an Ion Library Quantitation Kit (Life Technologies^TM^, United States), then concentrations were adjusted to 26 pM. Amplicons were attached to Ion Sphere Particles using Ion PGM Template OT2 400 kit (Life Technologies^TM^, United States). Sequencing was performed with Ion Xpress Barcode Adapters (1-16 Kit; Life Technologies^TM^) and a 318^TM^ chip (Life Technologies^TM^) on an Ion Torrent Personal Genome Machine (Life Technologies^TM^). Sequences were binned by sample and quality filtered within the PGM software (Torrent Suite^TM^ software life Technology) to remove polyclonal and low-quality reads. Fastq files for each sample were exported for the subsequent bioinformatics analysis.

#### High-Throughput Sequence Analysis

The quality and number of reads for each sample were assessed using FASTQC v0.11.4^1^. Raw sequences were filtered by quality using scripts from FASTXToolkit^2^. Only reads where at least 80% of the sequence had a minimum acceptable Phred quality score of > 20 were retained. Reads which passed all quality control steps were concatenated into a single FASTA file for subsequent processing. Filtered quality sequences were analyzed using the Quantitative Insights Into Microbial Ecology (QIIME) software version 1.8.0 (Caporaso et al., 2010b). Sequences were clustered in OTUs using a 97% sequence similarity threshold using open-reference OTU picking approach with USEARCH pipeline version 6.1 (Edgar, 2010). This pipeline involves clustering, chimera checking, and quality filtering. The taxonomy was assigned using RDP classifier (Wang et al., 2007) and Greengenes database gg_13_8_otus (DeSantis et al., 2006). The OTUs representative sequences were aligned using Pynast (Caporaso et al., 2010a) with a minimum sequence length threshold of 150 bp. A phylogenetic tree was constructed with FastTree (Price et al., 2010) Finally, in order to reduce artifactual sequence, the resulting OTU table was filtered at 0.005% to remove singletons (OTUs represented by only a single sequence) and reduce spurious OTUs (Navas-Molina et al., 2013; Flynn et al., 2015). In addition, reads belonging to Streptophyta were removed from the dataset and not included in the analysis as members assigned to this taxon were considered to be contamination of chloroplast from diet and water, and not a part of the gut microbiota (Rud et al., 2017). The core microbiota was calculated in QIIME and was defined as the OTUs shared in 100% of all the samples. Only mucosa samples were used for evaluating the core microbiota. The digesta samples were not included in this analysis due to the pooling of samples by tank. A Venn diagram representing the core microbiota was constructed in http://bioinfogp.cnb.csic.es/tools/venny/index.html (Oliveros, 2007).

Diversity metrics analyses were performed in QIIME rarefying all the samples at a depth of the lowest number of sequences (6.435 reads) across the samples. Alpha diversity of each sample was calculated using three metrics: Shannon, and whole-tree phylogenetic diversity (PD). Beta diversity was determined between samples with weighted and unweighted UniFrac (Lozupone and Knight, 2005). PCoA plots from beta diversity results were visualized with EMPeror (Vázquez-Baeza et al., 2013). Although some analyses were conducted to highlight major differences in the bacterial composition between digesta and mucosa, the comparisons were mainly focused on evaluating the differences between control and probiotic groups during the freshwater and seawater stages for both sample types independently. This was due to the differences in the sampling protocol between the digesta (pooled samples per tank) and the mucosa (individual samples per fish).

### Intestinal Gene Expression

Distal intestine from five fish during the seawater stage, i.e., 2 from two tanks and 1 from the third tank (*n* = 5) were sampled for gene expression analysis. Each target gene was normalized using the geometric average expression of two reference genes (elongation factor 1 and beta-actin). The primer sequences of the genes evaluated in this study are listed in Table 2.

**TABLE 2:** List of primers used for the gene expression analysis in the present study.

**Gene**	**Primer sequence (5′–3′)**	**Annealing temperature (°C)**	**Primer efficiency**	**Amplicon size (bp)**	**GenBank number**
*ef-1a*	F-TCTTGGTCGTTTTGCTGTGC R-AGCCTTGATGACACCGACAG	60	1.8	61	AF321836
*actin*	F-TCAGGGAGTGATGGTTGGGA R-GCCACTCTCAGCTCGTTGTA	60	2.0	171	NM_001123525.1
*tnf–a*	F-ACACACTGGGCTCTTCTTCG R-GCACTTGACCCTAAACGAAGC	58	2.0	52	NM_001123589.1
*pcna*	F-ACAGTTGTGTGGTCAGGATGC R-GAACTTAACGCCATCCTTGG	60	1.9	110	BT056931
*hsp-70*	F-TGGTCCTGGTGAAGATGAGG R-TGGCCTGTCTCTGTGAATCG	60	1.9	108	AJ632154
*tlr-3*	F-CTCTAACGGCAACCAGAAGC R-ATGGTGAGGTTGGACAGAGG	60	2.0	144	BK008646
*mx-1*	F-AAGCTGGCAGAGACACATGC R-ACATCCTTTCTGCCGAGTCC	60	1.9	73	NM_001123693

#### RNA Extraction and cDNA Synthesis

RNA from DI sections was extracted using TRI reagent (Sigma-Aldrich, Poole, United Kingdom) according to the manufacturer’s instructions, with some modifications as described by Rawling et al. (2019). To remove any contaminating genomic DNA, the RNA was treated with Dnase (TURBO DNA-free^TM^, Ambion) following the manufacturer’s instructions. The yield and quality of RNA in each sample was determined by measuring 260/280 nm and 260/230 absorbance ratios (NanoDrop Technologies, Wilmington, DE, United States). The integrity of RNA was confirmed by running the RNA extracted from the samples in a 1% agarose gel. RNA samples were stored at −80°C. A total amount of 1 μg of RNA was used for cDNA synthesis, using iScript^TM^ cDNA synthesis kit (Biorad, Berkeley, CA, United States) following the manufacturer’s instructions. For each set the samples, a negative control was included by performing a reaction with a pool of randomly selected RNA from samples of each experiment without the reverse transcriptase enzyme to control genomic DNA contamination. The synthesized cDNA and negative controls were diluted in molecular grade water and stored at −20°C.

#### Primer Optimization

All the primers for gene expression were synthesized by Eurofins MWG (Ebersberg, Germany). Primers sequences were designed using Primer3 (Rozen and Skaletsky, 1999) or obtained from previous publications. Primers specificity for reference and target genes were evaluated *in silico* by the tool Primer-BLAST (Ye et al., 2012) available at http://www.ncbi.nlm.nih.gov/tools/primer-blast/. Specificity was also checked by a melting curve after each qPCR assay and subsequent agarose gel electrophoresis to confirm the amplification of a single product with the expected molecular size and absence of primer-dimers. Amplification efficiency (*E*) was determined for each primer set using a standard curve based on five dilution series from cDNA (1:4 or 1:10), which were prepared by pooling an equal amount of cDNA from a representative number of samples from the same intestinal region. Each dilution was run in triplicate, and linear regression of the standard curve was constructed with quantification cycle (C_q_) values; R-squared (*R*^2^) and slope were also calculated. The amplification efficiency was calculated with the formula: (*E* = 10(^1/–slope^) − 1). *R*^2^ values and *E* for all primer sets were > 0.97 and 1.83–2.04, respectively.

#### Quantitative Real-Time Polymerase Chain Reaction (qPCR)

All Quantitative real-time PCR (qPCR) reactions were performed with the SYBR green method using a StepOne Plus^TM^ Real-time PCR thermal cycler (Applied Biosystems, Life Technologies) and with the QuantStudio^®^ 12K Flex Real-Time PCR system (Applied Biosystems, Life Technologies). Duplicate qPCR reactions were set on 384-well or 96-well plate by mixing 2.0 μl of cDNA template (1/10 or 1/20 dilution according to the experiment), 3.75 μl iTaq^TM^ Universal SYBR^®^ Green Supermix (Bio-Rad, Berkeley, CA, United States), 0.225 μl of forward and reverse primer (0.3 μM) and 1.3 μl of molecular grade water (Ambion). The thermal profile for all reactions was 10 min at 95°C and then 40 cycles of 15 s at 95°C, 60 s at 60°C. Fluorescence monitoring occurred at the end of each cycle, and additional melting curve analysis was performed using a temperature range of 60°C to 95°C at 0.3°C intervals. For each set of samples and gene evaluated two controls were used. First, a no-template control to ensure the absence of DNA contamination in the reagents and environment and second, a no reverse transcription control prepared during cDNA synthesis as previously described. The raw C_q_ values for reference and target genes were exported to Microsoft Excel and corrected by qPCR efficiency. Reference genes were chosen by ranking them according to overall coefficient variation and their interspecific variance as described by Kortner et al. (2011). Gene expression for each gene was normalized to the geometric average expression of two reference genes (elongation factor 1 and beta-actin) using corrected raw C_q_. Normalized gene expression of each target gene was calculated from corrected raw C_q_ (Pfaffl, 2001).

### Intestinal Histology

Histology was performed on fixed distal intestinal sections from three fish per tank from each sampling point (*n* = 9). Samples were dehydrated in graded ethanol concentrations prior to embedding in paraffin wax. For each specimen, multiple transverse sections (5 μm) were stained with haematoxylin and eosin (HE) as well as periodic acid–Schiff (PAS). Images from histology were taken from each DI section using a light microscope and analyzed with the Image J version 1.36 (National Institutes of Health, United States). The mucosa fold length average was measured in at least 15 well-oriented folds per section stained with HE. Fold length was only measured in primary folds with at a minimal length of 200 μm, complex folds were not taken into account. Goblet cells were counted in PAS stained sections and counted across a distance of 200 μm in at least fivefold per section and then averaged. The perimeter ratio (PR) of each intestinal section [arbitrary units (AUs)] was measured using the external perimeter (EP) and lumen perimeter (LP) and calculated by the formula: PR = IP/EP.

### Viral Challenge Post-seawater Transfer

An IPNV challenge was conducted using a virulent strain of IPNV [S-IPNV-TT96 isolate, serotype Sp (Johansen and Sommer, 2001)]. One passage away from primary isolation was used for the challenge. The virus isolate was propagated and quantified in Chinook salmon embryo-214 (CHSE-214) cells (Lannan et al., 1984) as described previously (Johansen and Sommer, 2001). Virus susceptibility was tested on the same batch of Atlantic salmon prior to challenge at SWT (start of week 7). After 6 weeks in FW, 2 × 30 fish (pan-jet marked to identify the tank of origin) from each experimental tank were transferred to SW in 2 tanks at the challenge unit. Three days post SWT, the fish were challenged through co-habitation by adding 20% IPNV infected cohorts (injected with 0.2 ml IPNV, dose 2,6 × 10^6^ infectious units per fish) to each tank. The temperature during the challenge trial was 10°C. Throughout the challenge, both tanks were fed the control SW diet. The tanks were monitored for 48 days following the challenge, with daily registrations of moribund/dead fish. The IPN status of dead fish was verified using an IPNV rapid co-agglutination (Hall et al., 1994) test developed by the National Veterinary Institute in Norway (Taksdal and Thorud, 1999). Verification of viral infection was also conducted by registrations of macroscopic signs of disease and isolation of IPNV from dead fish by titration of head kidney homogenates onto CHSE-214 cells in 96-well plates.

### Statistical Analysis

Statistical comparisons in all the analyses were conducted between the control and probiotic groups at the same sampling point, except in the LEfSe analysis where the effect of the SWT was evaluated comparing control groups between freshwater and seawater. To investigate experimental group differences between bacterial communities (beta diversity), the software package PRIMER-E v.6 PERMANOVA+ was used (PRIMER-E, Plymouth, United Kingdom) (Clarke and Gorley, 2006). Beta diversity was calculated in QIIME based on weighted and unweighted UniFrac metrics. Dissimilarity matrixes from UniFrac were imported to PRIMER-E to evaluate significant differences between groups by permutational multivariate analysis of variance (PERMANOVA). To assess alpha and beta diversity indexes of mucosa samples, a linear mixed model was applied using water habitat and diet as fixed factors, and tank as random factor. For digesta samples, a regular linear model (two-way ANOVA with water habitat and diet as fixed factors) was applied since samples were pooled per tank prior analysis.

Differences in the relative abundance of OTUs between groups were analyzed with LEfSe (Segata et al., 2011), available at http://huttenhower.sph.harvard.edu/galaxy/ using the default parameters. This tool first identifies significant differences among experimental groups and then evaluates whether these differences are consistent with other features such as the phylogenetic affiliation of the OTUs. LEfSe implements different statistics test involving firstly, a non-parametric factorial Kruskal–Wallis rank sum test; secondly, a pairwise test using Wilcoxon sum-rank test; and finally, linear discriminant analysis (LDA) to estimate the effect size of each differentially abundant OTU. To detect the impact of SWT on the potential differences of OTUs with LEfSe microbiota data from the control group was used.

Survival curves from the *in vivo* viral challenge were calculated by the Kaplan–Meier method and compared by log rank (Mantel–Cox) pairwise comparison testing for equality of survival pattern between groups using pooled replicates. Statistical analyses were performed using SPSS version 11.0 (SPSS, Inc., Chicago, IL, United States) with significance determined at *P* < 0.05.

The results of all other analysis (i.e., alpha diversity, gene expression, and histological evaluation) were analyzed using JMP Pro 14.3 (SAS Institute, Inc., Cary, NC, United States). The data was checked for normality (Shapiro–Wilk test) and homogeneity of variance (Tap et al., 2009). Data fulfilling parametric test assumptions was analyzed either by *t*-test or two-ways ANOVA (followed by Tukey HSD). Data that did not fulfill parametric test assumptions were log transformed to achieve normality or otherwise analyzed with a non-parametric test such as the Mann–Whitney *U*-test. All data are presented as mean ± standard deviation (SD), and significance was accepted at *P* < 0.05.

## Results

### Fish Performance

Growth performance was comparable between groups in both freshwater and seawater in terms of SGR and TGC (Table 3). The performance was judged in accordance with expectations for fish at this age and this type of diets.

**TABLE 3:** Fish growth performance.

	**FW^1^**		**SW^1^**	
		
	**Control**	**Probiotic**	**Control**	**Probiotic**
Initial body weight (g)	35.06 ± 0.17	34.88 ± 0.15	68.13 ± 2.66	67.03 ± 1.17
Final body weight (g)	67.88 ± 2.46	66.92 ± 1.14	144 ± 10.44	140 ± 5.57
SGR	1.57 ± 0.08	1.55 ± 0.05	1.92 ± 0.9	1.89 ± 0.11
TGC	1.60 ± 0.09	1.57 ± 0.06	2.35 ± 0.15	2.31 ± 0.15

*FW, freshwater; SW, seawater; SGR, specific growth rate; TGC, thermal growth coefficient. ^1^Data represent mean ± SD. No significant differences were detected between dietary groups.*

### Microbiota Analysis

#### High-Throughput Sequencing Data

A total of 5.0 million reads were produced from the 36 sequenced samples before quality control. After quality filtering, processing the data in QIIME, filtering spurious sequences and discarding reads belonging to Streptophyta, a total of 1,911,900 reads (53,108 ± 33,097 reads per sample) were retained. The percentage of removed reads belonging to Streptophyta was relatively low in digesta during FW, and mucosa during FW and SW (from 0.3 to 2.2% for probiotic and control groups, respectively), but much higher in the digesta in SW (i.e., 41.8 and 44.7% for probiotic and control groups respectively).

#### Intestinal Microbiota of the Distal Intestine

To assess whether the composition of the bacterial communities in the DI was influenced by SWT and the supplementation of the probiotic in the diet, several comparisons were performed using alpha and beta diversity metrics.

The rarefaction curve based on the Chao1 index reached the plateau (Supplementary Figure 1), suggesting that the sequencing depth had sufficient coverage to evaluate the diversity of both digesta and mucosa samples. Alpha diversity was evaluated using Shannon and PD indices (Table 4). There was a trend toward decreasing PD after SWT in both digesta and mucosa samples. This decrease in PD after SWT appeared more pronounced and statistically significant in the mucosa of the probiotic fed fish, suggesting a lower richness of low abundant OTUs associated with the mucosa of those fish. Shannon index, which takes into account species richness and evenness, showed differences between dietary treatments during SW in both digesta and mucosa samples. No effect of SWT on the digesta or mucosa of control fish was observed for the Shannon index. Nonetheless, probiotic fed fish showed a numerical trend toward decreasing Shannon diversity during SW. An opposite result was detected in the digesta of probiotic fed fish compared to control fed fish during SW. The alpha diversity results also indicated that the richness (PD) in the mucosa tend to be lower than in the digesta across all the experimental groups. In contrast, the Shannon index showed higher diversity in the mucosa compared to the digesta except in the probiotic group during SW.

**TABLE 4:** Alpha diversity of the distal intestinal microbiota comparing probiotic and control groups and SWT effect in the mucosa and digesta.

						**Two-ways ANOVA model/linear mixed model**					
						
	**Alpha diversity**					**Water**				**Water habitat ×**	
	**index^1^**	**FW**		**SW**		**habitat**		**Diet**		**Diet**	
						
**Tissue**		**Control**	**Probiotic**	**Control**	**Probiotic**	***f***	***P***	***f***	***P***	***f***	***P***
Digesta	PD	11.3 ± 1.1	10.4 ± 0.8	10.7 ± 0.6	9.4 ± 0.7	2.85	0.13	5.57	0.046	0.16	0.7
(*n* = 3)	Shannon^1^	5.3 ± 0.3ab	5.1 ± 0.2ab	5 ± 0.1a	5.8 ± 0.4b	0.99	0.35	3.50	0.010	10.8	0.011
Mucosa^2^	PD	9.4 ± 1.2a	9.9 ± 1.2a	8.8 ± 1a	6.5 ± 1b	3.71	0.002	1.93	0.126	35.87	<0.001
(*n* = 6)	Shannon	6 ± 0.2	5.9 ± 0.3	6.2 ± 0.4	5.2 ± 0.4	1.78	0.093	4.31	0.012	95.64	<0.001

*FW, freshwater; SW, seawater; PD, phylogenetic diversity. ^1^Values are means ± SD, letters are present when a significant difference was identified by two-ways ANOVA model and *post hoc* test (Tukey HSD). Means in a row without a common letter differ, *P* < 0.05. Alpha diversity was calculated from samples rarefied at an even depth of 6,435 sequences. ^2^A linear mixed model was used to evaluate statistical differences in bacterial microbiota of mucosa with Diet and Water habitat as fixed factors and Tank as a random factor.*

**FIGURE 1:**
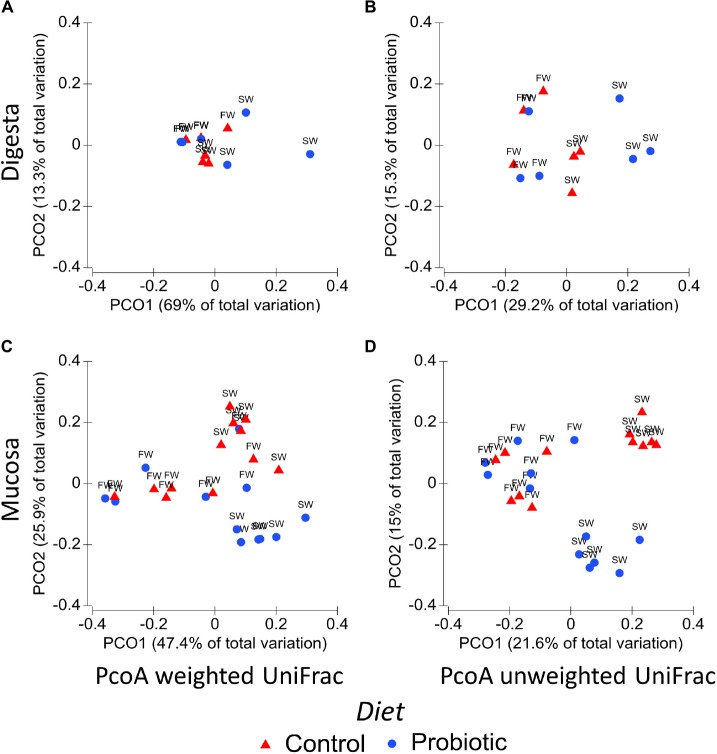
Principal coordinate analysis (PCoA) of the distal intestinal microbiota using UniFrac distances. The percentage of variation is explained by PC1 and PC2 axis. Each plot represents the differences between control (blue circles) and probiotic (red triangles) groups in both stages freshwater (FW) and seawater (SW). **(A)** PCoA weighted UniFrac digesta; **(B)** PCoA unweighted UniFrac digesta; **(C)** PCoA weighted UniFrac mucosa; **(D)** PCoA unweighted UniFrac mucosa.

Comparisons between experimental groups using weighted and unweighted UniFrac revealed substantial differences in the bacterial composition as demonstrated by PCoA plots and PERMANOVA analysis of the mucosa and digesta-associated microbiota (Figure 1 and Table 5, respectively). These results identified the water habitat, i.e., SWT, as the main driver of the differences in the bacterial composition in both digesta (weighted UniFrac Pseudo-F_1,8_ = 4.89, *P* = 0.012) and mucosa (weighted UniFrac Pseudo-F_1,4_ = 8.99, *P* = 0.001) Results from PERMANOVA analysis revealed an interaction between the factors diet and water habitat in mucosa-associated microbiota (weighted UniFrac, Pseudo-F_1,4_ = 4.68, *p* = 0.012). The latter is displayed in the PCoA plots of mucosa samples (Figure 1C) where there is an evident separation between control and probiotic samples in SW but not in FW. Significant differences between the control and the probiotic group in mucosa samples during SW were consistent in both weighted and unweighted UniFrac (Figures 1C,D) suggesting that the bacterial community between both groups not only differ in the presence and absence of some bacteria but also in the relative abundance of some taxa.

**TABLE 5:** PERMANOVA results from weighted and unweighted UniFrac.

	**PERMANOVA**							
	
	**Weighted UniFrac**				**Unweighted UniFrac**			
		
**Group comparison**	**Average dissimilarity**	**df**	**Pseudo-F**	***P***	**Average dissimilarity**	**df**	**Pseudo-F**	***P***
**Digesta**								
Diet	0.16	1	1.10	0.322	0.39	1	1.22	0.208
Water habitat	0.19	1	4.89	0.012	0.42	1	3.38	0.003
Diet × Water habitat		1	3.98	0.03		1	1.42	0.117
Residual		8				8		
**Mucosa^1^**								
Diet	0.37	1	3.78	0.032	0.58	1	2.18	0.015
Water habitat	0.39	1	8.99	0.001	0.61	1	6.52	0.001
Diet × Water habitat		1	4.68	0.012		1	3.19	0.002
Tank		4	1.13	0.361		4	0.91	0.677
Residual		12				12		

*^1^PERMANOVA analysis for mucosa used Diet and Water habitat as fixed factors and Tank as a random factor.*

**FIGURE 2:**
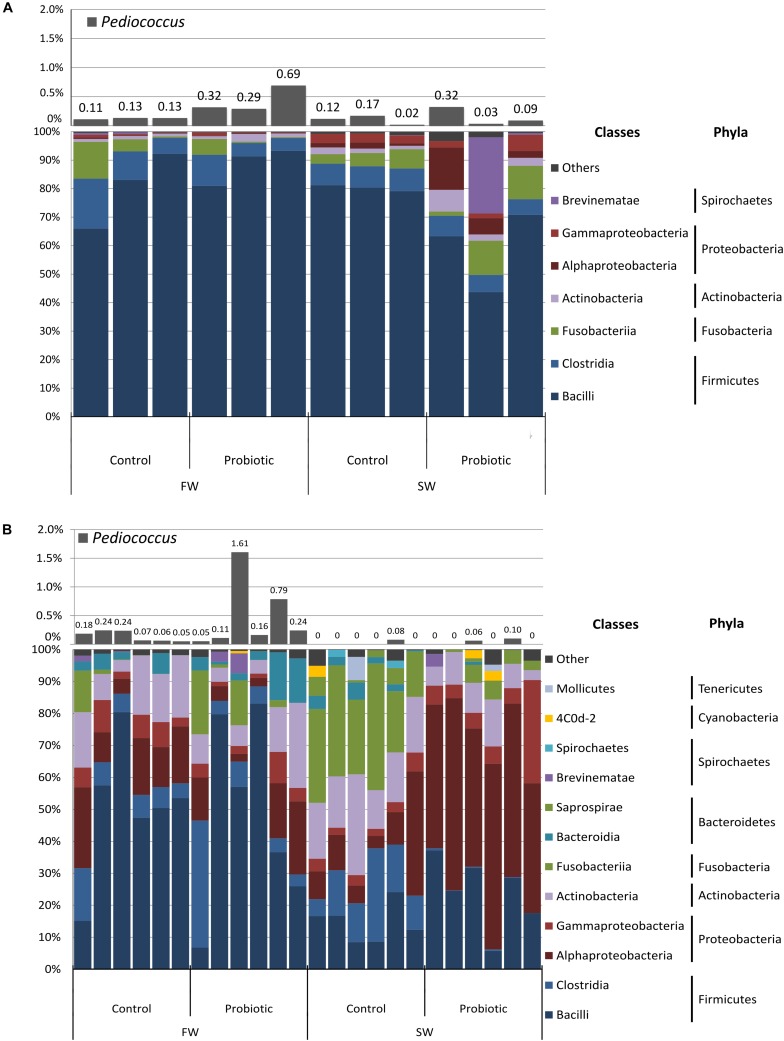
Relative abundance of bacterial communities at class and phylum level and abundance of the genus *Pediococcus* from **(A)** Digesta and **(B)** Mucosa of the distal intestine of Atlantic salmon fed control and probiotic diet during freshwater (FW) and seawater (SW) stages.

Five phyla in the digesta (*Firmicutes*, *Fusobacteria*, *Proteobacteria*, *Actinobacteria*, and *Spirochaetes*) and eight in mucosa (*Firmicutes*, *Proteobacteria, Actinobacteria, Fusobacteria, Bacteroidetes, Spirochaetes, Cyanobacteria*, and *Tenericutes*) accounted for more than 98% of the total abundance of the sequences derived from the samples (Figure 2 and Supplementary Table 1). The digesta samples were strongly dominated by the phylum *Firmicutes* (91.5 ± 7.3 and 95.3 ± 3% for the control and probiotic fed fish, respectively), mainly the classes *Bacilli* (>59%) and *Clostridia* (>6.2%). For the mucosa samples, the dominant taxa varied according to the water habitat and diet (Figure 2B). During FW the mucosa-associated bacterial microbiota was dominated by *Firmicutes* (57.1 ± 18.1 and 58.2 ± 24.4% for the control and probiotic fed fish, respectively), followed by *Proteobacteria* (23.6 ± 9.5 and 16.3 ± 12% for the control and probiotic group, respectively). In SW the most abundant phyla in the control group were *Firmicutes* (27.9 ± 8%), *Fusobacteria* (25.8 ± 9.3%), *Proteobacteria* (19.6 ± 13.1%) and Actinobacteria (17.7 ± 6.2%), whereas the most abundant phyla in the probiotic group were *Proteobacteria* (63 ± 8%), Firmicutes (22.7 ± 10.2%), and Actinobacteria (7.8 ± 3.4%). Hence the most striking differences between dietary groups were the higher abundance of *Proteobacteria* and the much lower relative abundance of *Fusobacteria* (2.2 ± 2.6%) in the probiotic group compared to the control group.

*Pediococcus* was identified by high-throughput sequencing in digesta and mucosa samples belonging to the probiotic fed fish in low relative abundance. The relative abundance of *Pediococcus* during the freshwater stage was 0.43 and 0.49% in digesta and mucosa, respectively, and 0.14 and 0.027% in digesta and mucosa in seawater (Figure 2). *Pediococcus* was also identified in the control group but at lower levels compared to the probiotic group, i.e., 3.4 times lower in digesta and mucosa during the freshwater stage and 1.45 and 2.05 lower in digesta and mucosa, respectively, during the SW stage.

**FIGURE 3:**
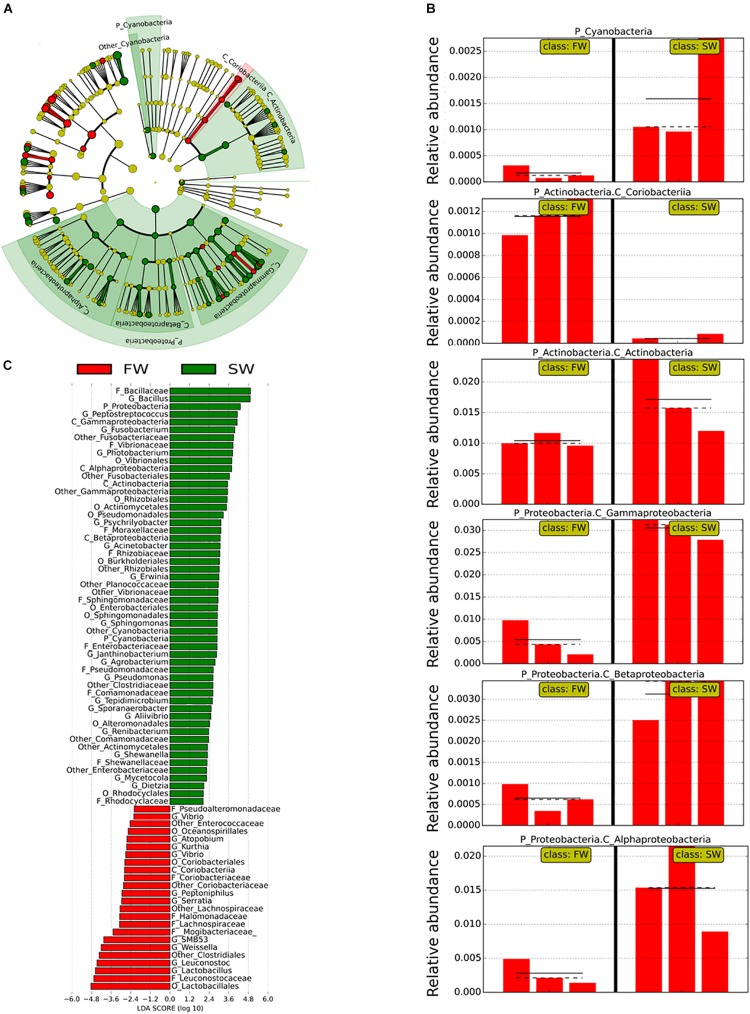
Taxonomic differences in the distal intestinal microbiota from digesta in control samples during FW and SW stages according to LEfSe analysis. The analysis was carried out with the relative abundance at the genus level. The stage (FW and SW) was treated as a Class. **(A)** A circular cladogram is representing the significant enriched OTUs between FW (red) or SW (green) groups. No significantly different OTUs are represented in yellow. The diameter of each dot is proportional to its effect size. **(B)** Relative abundance (expressed from 0 to 1) of enriched taxa according to LEfSe (only OTUs at class or phylum level were plotted). **(C)** Linear discriminant analysis (LDA), differentially enriched OTUs are arranged in descending order according to LDA score.

**FIGURE 4:**
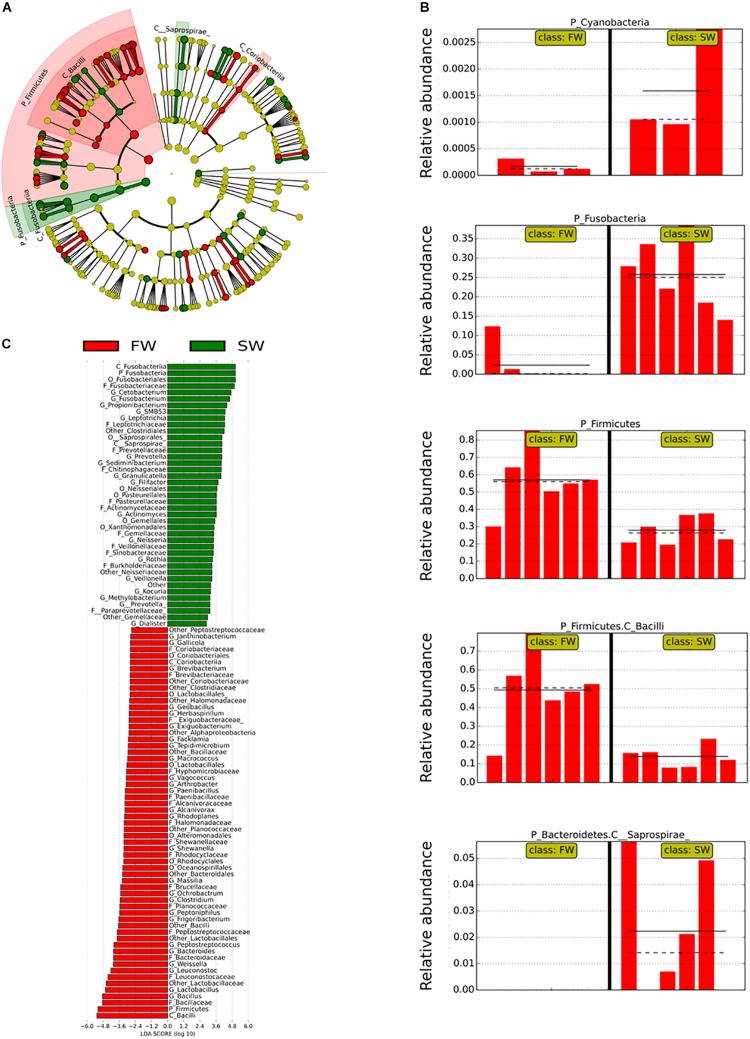
Taxonomic differences in the distal intestinal microbiota from mucosa in control samples during FW and SW stages according to LEfSe analysis. The analysis was carried out with the relative abundance at the genus level. The stage (FW and SW) was treated as Class. **(A)** A circular cladogram is representing the significant enriched OTUs between FW (red) or SW (green) groups. No significantly different OTUs are represented in yellow. The diameter of each dot is proportional to its effect size. **(B)** Relative abundance (expressed from 0 to 1) of enriched taxa according to LEfSe (only OTUs at class or phylum level were plotted). **(C)** LDA, differentially enriched OTUs are arranged in descending order according to LDA score.

Linear discriminant analysis effect size was used to identify the most important OTUs affected by the water habitat and the diet. To evaluate the effect of the SWT on mucosa- and digesta-associated microbiota, only samples from control groups in both stages FW and SW were compared (Figures 3, 4). A relatively high number of OTUs were observed to be affected by SWT in the digesta, mainly the genera *Bacillus*, *Fusobacterium*, and *Photobacterium* and several taxa from phylum *Proteobacteria* were the most significantly enriched during SW (Figure 3). Meanwhile, the most abundant taxa during FW belonged to the order *Lactobacillales* including the genera *Lactobacillus*, *Leuconostoc*, and *Weisella*. On the other hand, the main changes in mucosa-associated microbiota as a result of SWT were characterized by a relative decrease of the phylum *Firmicutes*, specifically the genera *Bacillus*, *Lactobacillus*, *Leuconostoc*, and *Weissella*. Whereas the phyla *Fusobacteria* (genera *Cetobacterium* and *Fusobacterium*) were the most significantly enriched taxa during SW (Figure 4).

**FIGURE 5:**
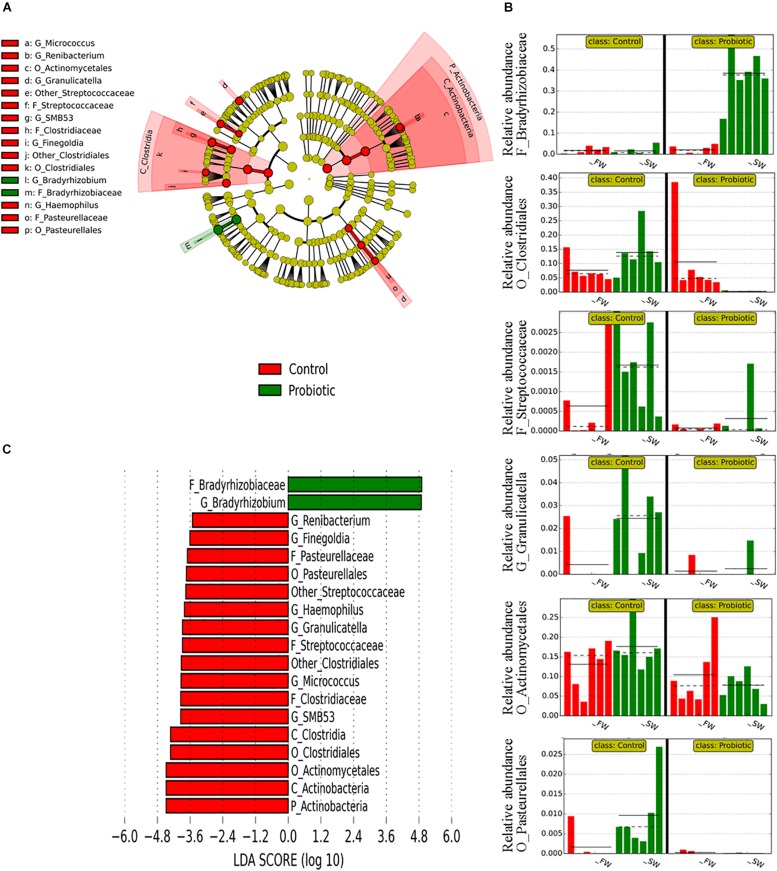
Taxonomic differences in the distal intestinal microbiota from mucosa between control and probiotic groups according to LEfSe analysis. The analysis was carried out with the relative abundance of all mucosa samples at the genus level. Control and probiotic groups were treated as classes, and FW and SW stages as subclasses. **(A)** A circular cladogram is representing the significant enriched OTUs between control (red) or probiotic (green) groups. No significantly different OTUs are represented in yellow. The diameter of each dot is proportional to its effect size. **(B)** Relative abundance (expressed from 0 to 1) of enriched taxa according to LEfSe. When more than one OTU from the same phylogenetic clade was enriched according to LEfSe, only the relative abundance of the closest phylogenetic ancestor was plotted. **(C)** LDA, differentially enriched OTUs are arranged in descending order according to LDA score.

**FIGURE 6:**
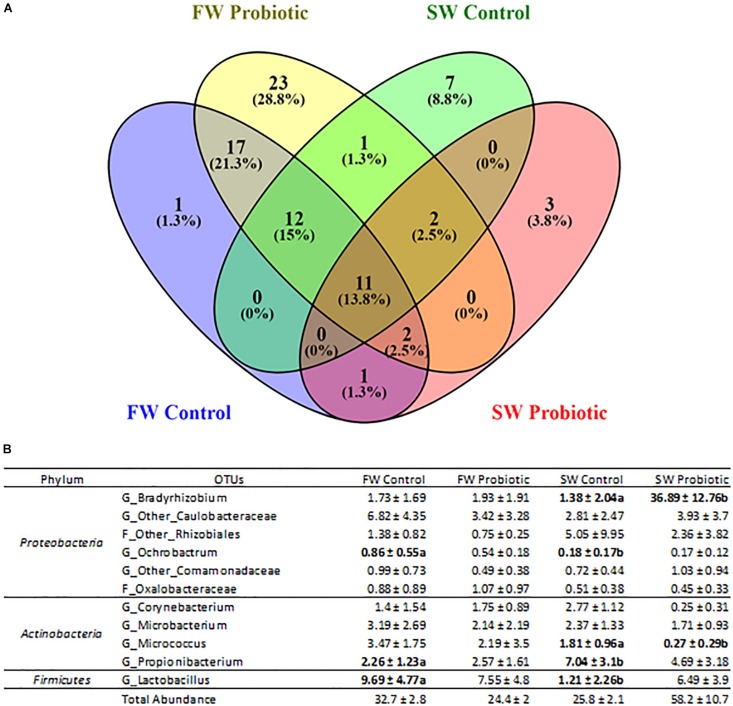
Core microbiota of distal intestinal mucosa. **(A)** Venn diagram showing the shared OTUs across 100% of the samples in all the experimental groups. **(B)** Relative abundance contribution for each of the OTUs belonging to the core microbiota of each experimental group. FW, SW stages. Values are means ± SD (*n* = 6), letters are present when a significant difference was identified by LEfSe.

Regarding the effect of probiotic diet on the microbiota, LEfSe results showed that the main differences between control and probiotic groups were observed in mucosa during SW (Figure 5). The microbiota of the probiotic fed fish seems to exclude a certain number of microorganisms after SWT, mainly members of phylum *Actinobacteria* and class *Clostridia*, while only one taxon with high relative abundance appeared to be significantly more represented in the probiotic fed fish (genus *Bradyrhizobium*, a group of non-pathogenic bacteria commonly encountered in water). In the digesta, only a few changes between probiotic and control fish were detected (Supplementary Figure 2) which is in agreement with the PERMANOVA results from UniFrac (Table 5). Only taxa with low relative abundances were significantly different between probiotic and control groups regardless of the water habitat (between 0.005 to 0.03% for the most abundant taxa, *Corynebacteriaceae*). The presence of a core microbiota regardless of water habitat and diet supplementation was determined only for the mucosa samples, as these were the only individual samples taken (digesta samples were pooled by tanks and thus not analyzed for core components) (Figure 6). A set of 11 common OTUs was shared in the probiotic and control groups during FW and SW. The shared OTUs belonged to the phyla *Proteobacteria* (6 OTUs), *Actinobacteria* (4 OTUs), and *Firmicutes* (1 OTU). The contribution of the core members in term of the relative abundance was similar in FW (ranged from 24.4 to 32.7%), however, in contrast during SW the contribution of core members varied from 25.8 to 58.2% from the control and probiotic groups, respectively. The most abundant core members during FW was *Lactobacillus* with a relative abundance that ranged between 9.69 and 7.55% for the control and probiotic groups, respectively. Meanwhile, in SW the most abundant core members were *Propionibacterium* (7.04%) in control group and *Bradyrhizobium* (36.89%) in the probiotic group. Three members of core microbiota were significantly modulated by SWT (*Ochrobactrum*, *Propionibacterium*, and *Lactobacillus*). Finally, probiotic supplementation affected the abundance of two taxa identified as core microbiota (*Bradyrhizobium* and *Micrococcus*).

### Gene Expression in the Distal Intestine

**FIGURE 7:**
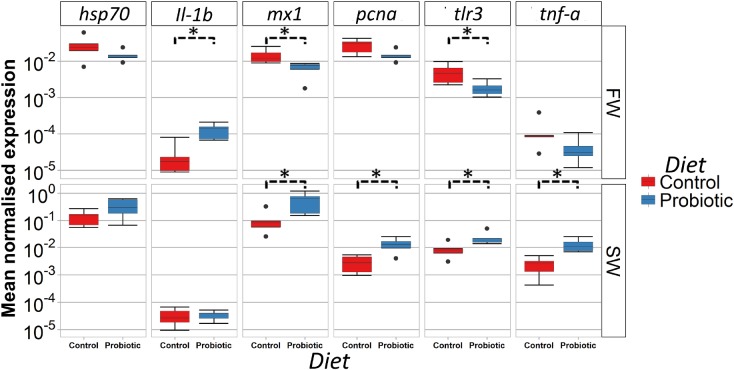
Gene expression profile (relative mRNA level) of the distal intestine of Atlantic salmon fed control and probiotic diets during FW and SW stages. Statistical differences between control and probiotic group (*n* = 5) (^∗^*P* < 0.05).

The expression of a panel of immune, stress, and apoptosis related genes in the DI was measured during FW and SW stages to evaluate the effect of the probiotic diet compared to the control diet (Figure 7). Genes related to antiviral protection were modulated in FW and SW by the probiotic diet; *mx1* and *tlr3* levels were lower in fish fed the probiotic diet in FW, but higher in the SW in comparison with fish fed control diet. Further, *pcna* and *tnfa* were significantly higher in the probiotic group than in the control group in seawater. A higher expression of *il-1b* was seen in the fish fed the probiotic diet in FW while *hsp70* was not affected by the probiotic treatment regardless of the water habitat.

### Histology

The parameters evaluated by light microscopy were not significantly different between the control fish and the probiotic fish (Table 6). The histological evaluation of distal intestinal morphology in control and probiotic groups during both FW and SW did not show any signs compatible with an active inflammatory response. The histological structure was characterized by a finger-like mucosal fold architecture, covered with an aligned epithelium of a single layer of enterocytes with supranuclear vacuoles in the apical zone, a thin lamina propria and low abundance of intraepithelial leukocytes suggesting a good health status of the fish.

**TABLE 6:** Histological parameters of the distal intestine of Atlantic salmon fed the experimental diets during freshwater and seawater stages.

	**FW**		**SW**	
		
	**Control**	**Probiotic**	**Control**	**Probiotic**
Mucosa fold length (μm)	322 ± 44	416 ± 58	518 ± 81	454 ± 106
Perimeter ratio (AU)	5 ± 1	6 ± 1	5 ± 1	6 ± 2
Goblet cells (per 100 μm)	16 ± 4	13 ± 3	16 ± 4	15 ± 3

*FW, freshwater; SW, seawater; AU, arbitrary units. Data represent mean ± SD (*n* = 9). No significant results were observed between dietary groups.*

### Viral Challenge

**FIGURE 8:**
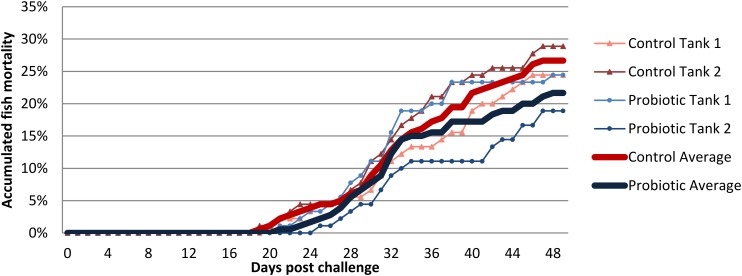
Accumulated mortality of probiotic and control fed fish challenged with IPN virus. Thin lines represent the mortality of each tank replicate and the average mortality in each group is displayed as a thick line.

The first IPNV related mortalities were registered in fish from the control group at 19 and 20 days post-challenge (in the two replicate challenge tanks), while the first mortality in the probiotic group was registered at 21 and 25 days post-challenge (Figure 8). Average cumulative mortality at termination (day 49) was 26.8 ± 3.2% for the control group and 21.7 ± 2.9% for the probiotic group. These differences in mortality were not statistically significant.

## Discussion

### Effect of Seawater Transfer on the Distal Intestinal Microbiota

The use of molecular methods such as high-throughput sequencing has rapidly expanded our knowledge of the bacterial communities in the fish intestine (Zhou et al., 2014; Tarnecki et al., 2017). In the present study, the main phyla observed in the DI of Atlantic salmon during both freshwater and seawater stages were *Firmicutes*, *Proteobacteria*, *Fusobacteria*, and *Actinobacteria*. Previous studies investigating the intestinal microbiota of Atlantic salmon under farming conditions have also found these phyla as normal residents of the intestine (Zarkasi et al., 2014; Gajardo et al., 2016, 2017; Schmidt et al., 2016; Dehler et al., 2017a; Lokesh et al., 2018; Rudi et al., 2018). Despite heterogeneous experimental conditions and approaches among studies, the phyla *Firmicutes* and *Proteobacteria* were consistently reported as dominant bacteria in the intestine of Atlantic salmon. Although some of the phyla were consistently observed in the DI of fish throughout this study, the SWT had a major role in modulating the abundance of specific bacterial communities (significant effect on four phyla: *Firmicutes*, *Fusobacteria, Proteobacteria*, and *Cyanobacteria*) at the digesta and mucosa level of the DI as evidenced by the LEfSe and beta diversity results. However, our results on the digesta microbiota must be cautiously interpreted since the pooling of 3 fish per sample may have impacted the possibilities to detect differences.

Even though several observations from the study of Dehler et al. (2017b) were based on data from digesta, our results are in agreement with their findings on the important role of SWT on modulating the intestinal microbiota of Atlantic salmon. Another important finding detected in both studies is the significant decrease in the relative abundance of several LAB genera after SWT. On the other hand, some important differences should be noted between the two studies, which could suggest the importance of considering both digesta- and mucosa-associated microbiota in such studies. In particular, we detected a strong increase in the anaerobic bacteria *Fusobacterium* and *Cetobacterium* in the mucosa, suggesting a potential strong contribution of *Fusobacteria* in Atlantic salmon during SW stages which was not observed in the study of Dehler et al. (2017b). *Fusobacteria* is often mentioned as part of oral and intestinal microbiota in humans (Chen and Jiang, 2015; D’Argenio and Salvatore, 2015). Some reports associate the presence of *Fusobacterium* spp. with different human pathologies (Kostic et al., 2012; Han, 2015). However, in fish, its clinical relevance remains poorly described. *Cetobacterium* has often been reported to be part of the gut microbiota of several fish species and also reported as a species with the potential to produce vitamin B12. Furthermore, Brugman et al. (2009) reported that a vancomycin treatment in zebrafish with enterocolitis increased the abundance of *Cetobacterium somerae*, which was associated with a reduction of inflammation.

We did not detect a strong contribution of *Mycoplasma* spp. (phylum *Tenericutes*) in the digesta of SW fish, as opposed to several other studies (Holben et al., 2002; Abid et al., 2013; Llewellyn et al., 2015; Jin et al., 2019) that have detected this genus as a substantial contributor of Atlantic salmon microbiota in SW and even as a putative member of the core microbiota (Dehler et al., 2017b). The reasons behind this result are not clear but the reported impact of environmental factors, dietary regimes, sampling strategy, and host genetic variation on the intestinal microbial communities of fish may explain the differences observed between studies (Tarnecki et al., 2017).

To the authors’ knowledge, our study is the first to report the effect of SWT on the mucosa-associated microbiota of Atlantic salmon. In both fish and mammals, it has been recognized that the digesta and mucosa compartments harbor substantially different microorganisms (Eckburg et al., 2005; Looft et al., 2014; Gajardo et al., 2016, 2017; Lyons et al., 2017). Thus, these two different microbial communities may have different roles in the intestine and may differently impact the health of the host. In fish, most of the studies investigating factors that modulate the gut microbiota have so far focused on the so-called allochthonous microbiota, which is associated with fecal or digesta samples. However, in mammals, several authors have speculated that the mucosa-associated microbiota could have a central role in modulating the intestinal physiology of the host. This hypothesis is based on the fact that the mucosa-associated bacteria could interact with the host epithelium both directly and indirectly, while those in the digesta only interact with the host indirectly (as reviewed by Van den Abbeele et al., 2011). The latter would suggest that the intestinal microbiota shift caused by SWT could have large implications for the gut health of salmon following SWT, hence supporting the particular interest to further study the impact of certain taxa associated with the mucosa, such as members of *Fusobacteria*, on salmon health. Prior to and during SWT commercially raised Atlantic salmon undergoes important physiological changes (i.e., smoltification) and is exposed to changes in salinity and to dietary modifications implemented to meet the nutritional requirements of the new smolt stage. Thus, this study did not aim to identify the weight of every single factor separately but highlight the extent of the impact of all the factors together on the intestinal microbiota under conditions that resemble the current commercial practices of Atlantic salmon production.

Salinity is a well-known factor affecting microbiota by limiting or promoting the establishment of specific bacterial communities in given environments (Lozupone and Knight, 2007; Canfora et al., 2014). The effect of salinity and SWT on bacterial communities associated with fish has been previously studied (Schmidt et al., 2015; Lokesh and Kiron, 2016; Lokesh et al., 2018; Rudi et al., 2018; Jin et al., 2019). A study conducted by Zhang et al. (2016) even demonstrated that the bacterial community associated with the fish intestine also respond to salinity changes. Nevertheless, the changes in intestinal microbiota during SWT is most likely due to the combination of several factors that will lead to a competitive niche appropriation by certain microbial species hence reorienting the bacterial composition in the gut. In particular, the host as “habitat filter” influenced by the shift in osmoregulatory functions of the gut and the altered immune response after SWT (Johansson et al., 2016; Karlsen et al., 2018). Based on our results we propose using SWT experiments, coupled with meta-transcriptomic and metabolomics analysis, to assess the functional role of the intestinal mucosa-associated microbiota in salmon during FW to SW transition.

### Effect of Probiotic on Distal Intestinal Microbiota

The use of probiotics in aquaculture has been implemented as a regular practice to improve the health and performance of fish under stressful farm conditions (Lauzon et al., 2014; Pérez-Sánchez et al., 2014; Hoseinifar et al., 2018, 2019). The beneficial effects of some probiotics in humans and animals are well-documented, but more research is necessary to ascertain their modes of action. In aquaculture, this limitation is also evident. Some of the proposed possible modes of action of the probiotics are suggested to be mediated by the modulation of the microbiota (Merrifield and Carnevali, 2014; Wang et al., 2015). The present study investigated the effect of the only dietary probiotic currently registered for use in aquaculture in the European Union, namely *P. acidilactici* MA18/5M. The scientific literature about the use of this specific probiotic strain in aquatic animals is continuously increasing (Castex et al., 2008; Ferguson et al., 2010; Merrifield et al., 2011; Lamari et al., 2013, 2016; Standen et al., 2013). Currently, two published studies reported the role of this probiotic in the intestine of Atlantic salmon during the seawater stage (Abid et al., 2013; Vasanth et al., 2015). Both studies were able to identify a positive effect of this bacterium in the intestine of Atlantic salmon; however, only the study from Abid et al. (2013) investigated the effect on the intestinal microbiota. In the present study, the genus *Pediococcus* was detected by high-throughput sequencing in digesta and mucosa samples from both groups, supporting the fact that *Pediococcus* is a ubiquitous genus encountered in the intestinal microbiota of salmonids. Indeed this genus has been previously reported as a normal inhabitant of the gut microbiota of Atlantic salmon (Merrifield et al., 2014; Gajardo et al., 2016; Dehler et al., 2017b) and rainbow trout (Araújo et al., 2016). In our study, our sequencing strategy did not allow to differentiate autochtonous *Pediococcus* species/strains from the one used in the probiotic diet. Nevertheless, the *Pediococcus* genus was detected at a higher level in samples from probiotic fed fish compared to the control group. Focusing on the probiotic group, overall the abundance of this genus was low in both water habitats as previously established by our group (unpublished data), with the higher relative abundance in the digesta of FW fish and the lowest abundance observed in the mucosa samples during the seawater stage. Lower abundance of *P. acidilactici* in mucosa compared with digesta in Atlantic salmon is in line with the study from Abid et al. (2013). Other studies using *P. acidilactici* as a probiotic in fish have demonstrated that this bacteria was able to survive in the intestine of freshwater (Ferguson et al., 2010; Merrifield et al., 2011) and seawater fish (Villamil et al., 2010; Lamari et al., 2013). Remarkably, despite this lower abundance, the strongest modulation of the microbiota in fish fed the probiotic was observed during the seawater stage in the intestinal mucosa. In fact, the probiotic effect on the mucosa-associated microbiota was an important factor driving the differences between dietary groups as shown by beta diversity results. This finding could support the hypothesis of a strong contribution of a probiotic induced “host effect” driving the different clustering of the microbiota composition. However, the significant interaction with water habitat revealed by the PERMANOVA analysis from UniFrac suggests that the probiotic effect is dependent on the water habitat. The most striking changes detected in the probiotic group at the mucosa level consist in the modulation of the relative abundance (↑ increase or ↓ decrease) of taxa belonging to class *Fusobacteriia (↓)*, orders *Clostridiales (↓)*, *Actinomycetales (↓), Pasteurellales (↓), and family* Bradyrhizobiaceae *(↑) and Streptococcacea (↓).* Interestingly, apart from Bradyrhizobiaceae that were more predominant in the mucosa, the microbiota of probiotic fed fish seems to display an “antagonistic like pattern” among several taxa. Some of these taxa (*Streptococcacea* and *Pasteurellales* for instance) are known as pathogenic or opportunistic bacteria in fish. Therefore, a reduction in their abundance at the mucosa level can be seen as a positive sign, but the absence of information about the other taxa affected by the probiotic (*Actinomycetales* and *Fusobacteriacea* in particular) does not allow us to draw further conclusions. Information about the function of these bacteria in the intestine and the role in the adaptation of Atlantic salmon to seawater require further investigation. Finally, most of these changes were only observed in the mucosa, highlighting the importance to investigate such samples when assessing the effect of probiotics in fish.

### Core Microbiota

Previous studies in salmonids have implied the presence of a so called core intestinal microbiota based on next generation sequencing of digesta or mucosa samples (Wong et al., 2013; Gajardo et al., 2016, 2017; Dehler et al., 2017a; Rudi et al., 2018). In the present study, we investigated the core microbiota in the intestinal mucosa and despite the large effect of probiotic administration and SWT, a “core like” mucosal microbiota with 11 shared OTUs was identified across all samples regardless of the water habitat and diet. This set of “resilient” microorganisms account for at least 24% of the relative abundance of the bacterial sequences. This suggests that the OTUs identified as members of this core microbiota may play an important role in the DI of Atlantic salmon, for instance by fulfilling central functions in the gut. The relatively high number of OTUs belonging to the phylum *Proteobacteria*, as part of the core microbiota of Atlantic salmon is in agreement with previous studies (Gajardo et al., 2016, 2017; Dehler et al., 2017b), whereas the absence of *Tenericutes* and *Bacteroidetes* or presence below detection thresholds must be noted. Seawater transfer had a significant effect on the core microbiota member *Lactobacillus* which was significantly lower in the control group in SW. It is important to highlight that *Lactobacillus* has previously been identified as a member of the core microbiota in SWT-related studies (Dehler et al., 2017b; Rudi et al., 2018), this finding deserves further investigation to clarify the role of this well-adapted LAB in the intestine of Atlantic salmon. Fish fed the probiotic diet had the strongest effect on the abundance of different taxa of the core microbiota, particularly in the genus *Bradyrhizobium* which had a relatively low abundance in control and probiotic group during FW 1.73 ± 1.69 and 1.93 ± 1.91% respectively but was significantly more abundant in the probiotic group in SW (36.89 ± 12.76%) compared to the control group (1.38 ± 2.04%).

### Effect of Probiotic on Gene Expression Profile and Antiviral Resistance

Probiotic administration influenced the expression of some of the genes investigated in this study, however, the type of response tended to differ according to the water habitat. Activation of *tnf*-α and *il-1b* in the intestine are commonly associated with stimulation of the immune response. In the present study, fish fed the probiotic diet had a significantly higher response in *il-1b* and *tnf*-α compared to fish fed the control diet. However, this response was only observed in FW. The activation of genes encoding pro-inflammatory cytokines in fish after supplementation with *P. acidilactici* has also been reported previously in Atlantic salmon (Abid et al., 2013; Vasanth et al., 2015) and tilapia (Standen et al., 2013). These authors suggested that activation of pro-inflammatory cytokines after supplementation with *P. acidilactici* may indicate a potential immuno-stimulatory response that could be beneficial to fight an eventual pathogen aggression.

Previous studies have investigated the expression of *hsp70* and *pcna* as markers for intestinal stress and cell proliferation in Atlantic salmon after adverse intestinal conditions (Olsvik et al., 2007; Sanden and Olsvik, 2009; Krogdahl et al., 2015). In this study, the low differences in the expression of both *hsp70* and *pcna* between the control and probiotic groups, together with a normal histological morphology of the intestine suggest that neither fish fed the control diet nor fish fed the probiotic diet were undergoing an inflammatory response in the intestine.

A decrease in the expression of several immune genes involved in the antiviral response has previously been reported in Atlantic salmon during the transition from freshwater to seawater (Johansson et al., 2016; Karlsen et al., 2018). In this study, the dietary supplementation with *P. acidilactici* MA18/5M modulated the intestinal antiviral response. This modulation was dependent on the water habitat as both investigated genes, namely *tlr3* and *mx1*, were significantly lower compared to the control the day before SWT, whereas a significant increase was observed after 5 weeks in seawater. This response to the probiotic has already been observed in SW (Abid et al., 2013) but not in FW. This may suggest that mechanisms associated with the water habitat are influencing the antiviral response and the response of the fish to the probiotic. One possible explanation for the different response of the intestine to probiotic supplementation could be related to the profound changes in the microbiota composition that take place during SWT. It is well-known that the intestine plays a major role in the adaptation to the new marine habitat. It is important to highlight that the strength of the modulation of *mx1* was higher during the seawater stage (five times increased compared to the control group) than in the freshwater stage (2.2 times decreased compared to the control group). Studies have demonstrated that *mx1* is expressed at high levels after the stimulation of *tlr3* agonist in head kidney leukocytes suggesting that these two genes are connected during the antiviral response (Arnemo et al., 2014). These results are in agreement with Abid et al. (2013) who demonstrated up-regulation of *tlr3*, *tnf*-α, and *mx-1* in the distal and proximal intestine of Atlantic salmon in SW stage (post-smolts) under a dietary regimen supplemented with *P. acidilactici* MA18/5M and short-chain fructooligosaccharides (scFOS) as synbiotic additives. The main function of *tlr3* in innate immunity is to act as a sensor of viral RNA. Moreover, some authors suggest that the activation of *tlr3* could trigger the expression of genes encoding cytokines and proteins responsible for modulating the immune response against viral infections (Rodriguez et al., 2005). Even though, *tlr3* expression in healthy Atlantic salmon has been detected in several tissues, the gut and spleen showed the highest level of expression (Arnemo et al., 2014). Due to the important role of these genes in antiviral response, their increase in expression in seawater may suggest a potential protective role of the probiotic in an eventual viral infection. In order to evaluate further the potential antiviral effect of *P. acidilactici* MA18/5M, an experimental challenge with IPN virus was conducted 3 days after SWT. The average accumulated mortality was lower in the fish previously fed the probiotic diet (during FW phase) compared to fish fed the control diet (22 and 27%, respectively), even though this difference was not statistically significant. The mentioned finding may suggest a potential role of the probiotics in improving anti-viral response. It is worth noting that the fish in the challenge tanks were fed the same control diet throughout the challenge. This means that the “probiotic” fish only had a history of being fed probiotics in FW. The results from the feeding trial part of this study, indicating a larger modulatory effect of the probiotic during SW, suggest that the variable results obtained in the challenge could be due to the lack of probiotics feeding during this experimental stage. Previous studies have looked at the resilience of *P. acidilactici* following a switch to a non-supplemented feed, showing that the probiotic bacteria is not detectable after 3 days in rainbow trout (Abid, 2014). This means the probiotic bacteria and its effect could have been present at least early in the challenge trial. Interestingly, the onset of mortalities started later for the “probiotic” fed fish relative to the control in both challenge tanks, and the probiotic group from the tank with the lowest cumulative mortality had a 6 day delay in this onset. As no samples for microbiota composition was taken from this part of the trial we cannot assess whether the probiotic was maintained in the gut of these fish longer than in the other tank. Ideally, the number of fish per tank and the number of replicate tanks should have been higher, in order to be able to detect potential differences. Additional future experiments should also allow for the continued feeding of the probiotic supplemented diet during the viral challenge. As such, further studies are necessary to confirm whether the induction of the antiviral response seen in this study as well as in Abid et al. (2013) results in a significant improvement in survival after viral infection.

## Conclusion

The factors evaluated in this study, i.e., dietary probiotic supplementation and transfer from freshwater to seawater, had a substantial impact on the bacterial communities of the DI of Atlantic salmon with a much more pronounced effect in the mucosa-associated microbiota. This result highlights the importance to consider both digesta and mucosa for future microbiota-based studies on the effect of SWT or dietary factors on fish. The changes in the mucosal microbiota in fish fed the probiotic supplemented diet in seawater suggest activation of the antiviral response. Future studies should be performed to investigate whether or not microbiota changes observed in this study have a causal link with the local immune changes and the implications this may have in term of salmon health.

## Data Availability Statement

The datasets generated for this study can be found in the BioProject ID: PRJNA546123 https://www.ncbi.nlm.nih.gov/bioproject/546123.

## Ethics Statement

The animal study was reviewed and approved by the project was approved by the Food and Safety Authority ID 4986: “The use of functional feed to improve the overall performance of Atlantic Salmon smolt.”

## Author Contributions

DM, MC, EA, JT, HM, and L-HJ: experiments design. HM and L-HJ: field experiments. AJ-T, MR, AR, and HM: sample collection. AJ-T, MR, and AR: laboratory experiments. AJ-T: bioinformatic analysis and writing the first draft of the manuscript. AJ-T, DM, MC, AR, L-HJ, and TF: data interpretation. TF, MC, AR, and DM: manuscript revision and editing. All authors read and approved the final version of the manuscript.

## Conflict of Interest

JT, TF, and EA are employed by BioMar AS and MC by Lallemand SAS. The remaining authors declare that the research was conducted in the absence of any commercial or financial relationships that could be construed as a potential conflict of interest.

## Footnotes

1 http://www.bioinformatics.bbsrc.ac.uk/projects/fastqc/

2 http://hannonlab.cshl.edu/fastx_toolkit
