# Supplementary material for: Influence of Dietary Supplementation of Probiotic Pediococcus acidilactici MA18/5M During the Transition From Freshwater to Seawater on Intestinal Health and Microbiota of Atlantic Salmon (Salmo salar L.)
Source: Front Microbiol. 2019 Sep 27;10:2243. doi: 10.3389/fmicb.2019.02243 (PMC6777325; doi:10.3389/fmicb.2019.02243)
Supplement: Supplementary file 1 [file Image_1.pdf]

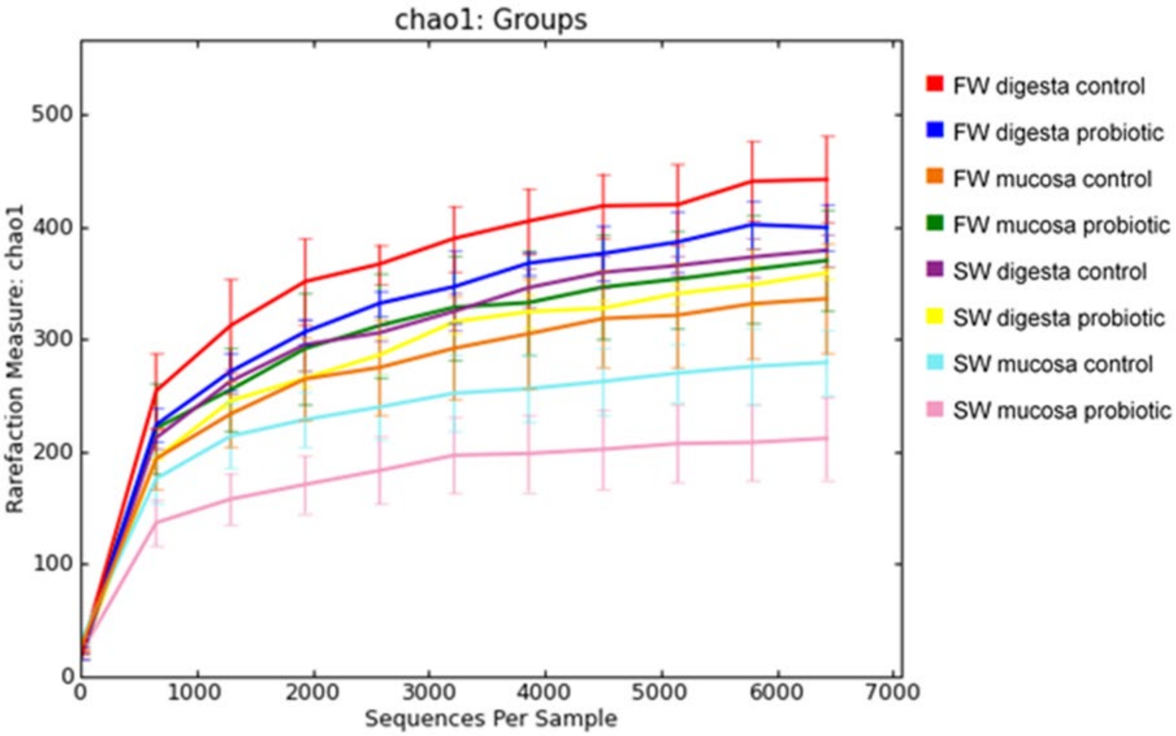

Supplementary Figure 1. Rarefaction curve based on Chao1 metric representing the average and standard deviation (error bars) of OTUs per experimental group

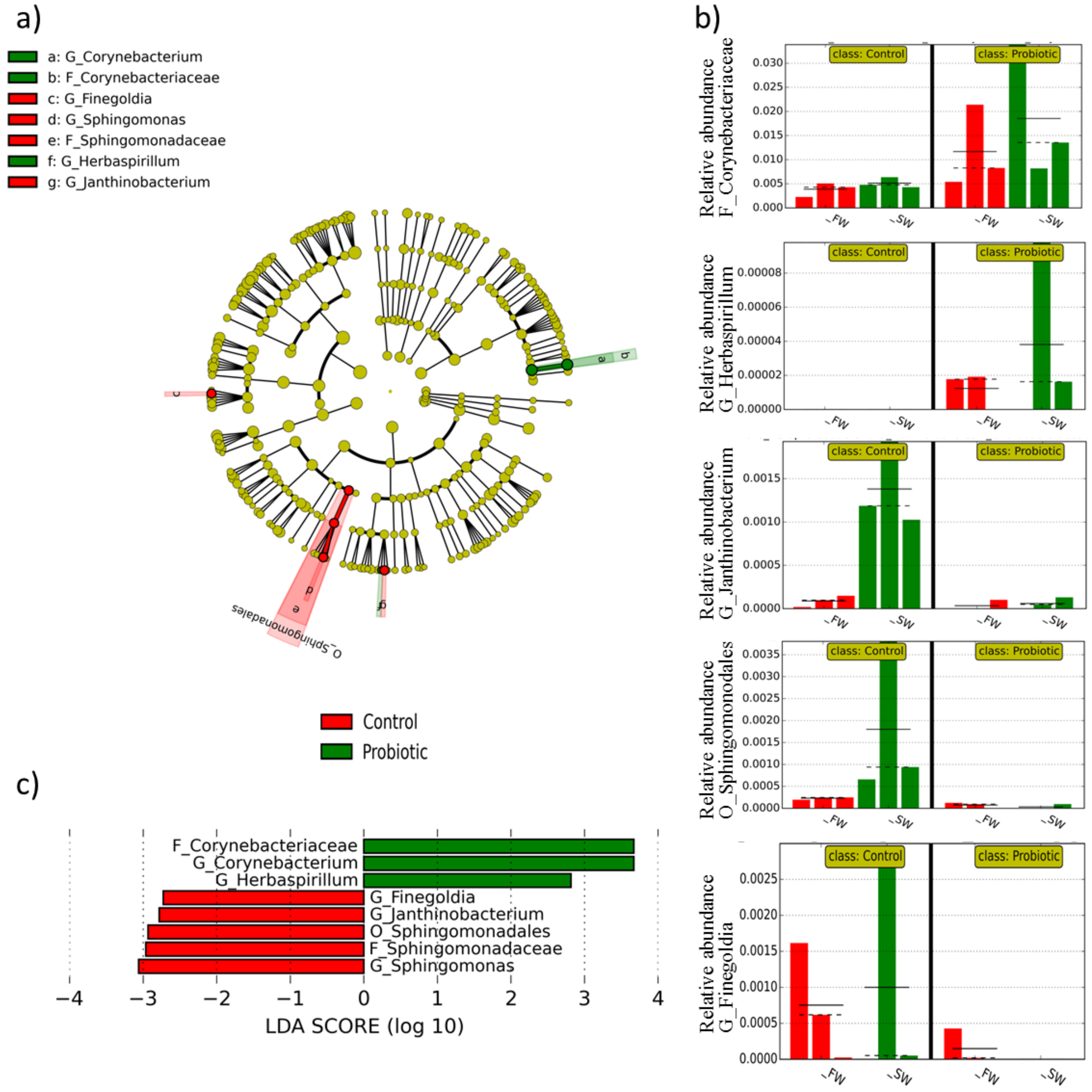

Supplementary Figure 2. Taxonomic differences in distal intestinal microbiota from digesta between control and probiotic groups according to LEfSe analysis. The analysis was carried out with the relative abundance of all digesta samples at the genus level. Control and probiotic groups were treated as classes and freshwater (FW) and seawater (SW) stages as subclasses. a) A circular cladogram is representing the significant enriched OTUs between control (red) or probiotic (green) groups. No significantly different OTUs are represented in yellow. The diameter of each dots is proportional to its effect size. b) Relative abundance (expressed from 0 to 1) of enriched taxa according to LEfSe. When more thoutan one OTU from the same phylogenetic clade was enriched according to LEfSe, only the relative abundance of the closest phylogenetic ancestor Was plotted. c) Linear discriminant analysis (LDA), differentially enriched OTUs are arranged in descending order according to LDA score.
